# Understanding chemical pathways of brown centre formation in laboratory induced and conventionally dried nut-in-shell macadamia kernels

**DOI:** 10.1016/j.heliyon.2024.e25221

**Published:** 2024-01-30

**Authors:** Marcela Martinez, Helen M. Wallace, Chris Searle, Brittany Elliott, Shahla Hosseini Bai

**Affiliations:** a Centre for Planetary Health and Food Security, School of Environment and Science, Griffith University, Nathan, QLD, 4111, Australia; b MacAvo Consulting, Pashley's Road, Welcome Creek, QLD, 4670, Australia

**Keywords:** Maillard reaction, Internal discoloration, Brown centre, Tree nuts, Sugars

## Abstract

World tree nut production has increased rapidly by around 50 % in the past decade; however, nut defects cause losses. For example, we know that brown centres are a major internal discolouration defect in macadamia nuts and are linked to the storage of nut-in-shell under improper conditions at high temperature and humidity. However, key chemical changes in brown centre kernels have not been described. In this study, we compared brown centres and white kernels from: 1) samples that were “induced” in the laboratory by storing at high moisture concentration; and 2) samples that were dried immediately after harvest using industry best practice methods recommended by the Australian Macadamia Society (AMS). We measured the moisture concentration, sugar concentration, fatty acid concentration, peroxide value, nutrient concentration and volatile compounds of induced and AMS samples. Our results showed that storing nut-in-shell macadamia under wet and hot conditions increased brown centres compared with samples immediately dried using the AMS regime, 10.33 % vs 1.44 %, respectively. Induced brown centres had significantly higher moisture concentrations than induced white centres. Volatile compounds including nonanoic acid, octanoic acid and 2,3 butanediol were identified and associated with brown centre formation in macadamia kernels and the initiation of lipid oxidation. Our results suggest sugar hydrolysis and the Maillard reaction are associated with brown centres both in laboratory induced samples and those formed using industry best practice drying methods. Our study suggests improper drying and storage at high temperature and high humidity are likely to result in brown centre formation. We recommend brown centre losses can be reduced by appropriate drying and storage practices.

## 1 Introduction

Tree nuts are a healthy food, that are rich in protein, dietary fibre, vitamin E, magnesium, copper, potassium and unsaturated fatty acids [[1], [2], [3]]. World tree nut production has increased by around 50 % in the past decade, due to increased demand for healthy foods, reaching values of 5.3 million tonnes in the 2020/2021 season and an annual global trade value of $USD 38 billion [4]. However, tree nuts are susceptible to multiple quality issues including lipid oxidation, free fatty acid formation, kernel immaturity, internal discolouration, insect attack and fungal contamination [[5], [6], [7]]. The term attributed with the internal discoloration of nuts differs depending on the type of nut, for example in almonds it is referred to as ‘concealed damage’, as ‘opalescence’ in pecans, and as ‘internal browning’ or ‘brown centres’ in both hazelnuts and macadamia nuts [[8], [9], [10], [11]]. Poor postharvest management practices, increased rainfall, elevated temperatures and poor drying practices have been associated with the occurrence of kernel discoloration [9,12,13]. However, high levels of kernel discoloration often occur even after appropriate postharvest management.

Internal discolouration is often only visible after kernel drying and roasting [10,14]. Oil oxidation, sugar hydrolysis and protein degradation are chemical reactions that produce compounds that may react to form brown colouration through the Maillard Reaction [10,15,16]. In roasted macadamia kernels, higher concentrations of reducing sugars have been associated with formation of brown colouration in kernels [17]. Elevated temperature, high moisture concentration and calcium deficiency have also been implicated in internal discolouration in many nuts [8,[10], [11], [12],^14^]. For example, almonds develop higher levels of concealed damage when roasted with high initial moisture concentrations compared with those roasted with lower initial moisture concentrations [10]. The drying process slows chemical reactions and is a recommended postharvest practice to stabilise nut products for long term storage [5]. Nutrient management is also a potential pathway for brown centre formation, as in some instances, the timely application of calcium to the soil has been associated with a reduction in brown centres in hazelnuts [8]. Brown centre formation after roasting has been investigated where kernels are subjected to high temperatures over 100 °C [14,18,19]. However, the nuts are dried and stored as nut-in-shell prior to being cracked for further processing [14,18]. Brown centre formation has been reported even after nut-in-shell drying and before roasting [9]. The main chemical drivers of brown centre formation after drying are not yet understood for many nuts when the nut-in-shell samples are dried in relatively low temperatures, i.e., under 60 °C.

Macadamia kernels are considered a gourmet food for their delicate flavour, crunchy texture and high oil content; however, they are susceptible to brown centre defects which costs Australian growers $USD 4.9 million per year [20]. Brown centre formation in macadamia kernels stored as nut-in-shell can be induced in the laboratory when they are maintained at high moisture of approximately 25 % concentration for five days or more at temperature above 30 °C [13]. High moisture concentration accelerates the breakdown of sucrose into reducing sugars that are needed for the Maillard reaction [9,12,21]. However, the chemical differences associated with browning in raw macadamia kernels, both in nut-in-shell samples kept at high moisture concentration and those produced from standard postharvest handling practices, have not previously been explored.

The mechanisms involved in brown centre formation of macadamia kernels particularly after roasting has been previously studied [14,16,17,22]. Our previous study has highlighted the nutritional and chemical differences between brown centre and white macadamia kernels where samples have been collected randomly with unknown origins [15]. However, the differences in brown centre chemistry and mechanisms explaining the brown centre occurrence developed under different postharvest conditions remained uncertain. This study aimed to compare the chemistry of the brown centre component of defect macadamia kernels with white macadamia kernels. In particular, we compared brown and white kernels from two sources: 1) nut-in-shell samples that were “induced” in the laboratory by storing at high moisture concentration; 2) nut-in-shell samples that were dried immediately after harvest using industry best practice methods recommended by the Australian Macadamia Society (AMS industry practice). We compared the moisture, sugar concentration, fatty acid concentration, peroxide value and nutrient concentration of macadamia kernels. We used kernels from a single cultivar and site to exclude differences due to genetics or management practices in different orchards. The results of this study can be used to determine the chemical changes that occur in brown centre kernels and give insights into ways that this costly quality issue can be managed.

## 2 Materials and methods

### 2.1 Treatment of nut-in-shell macadamia samples and classification of brown centres

Macadamia nut-in-shell (NIS) samples of variety 849 were collected from an orchard in the Bundaberg region, Queensland in 2021 and transferred to Griffith University within 24 h after harvest and de-husking. Nut-in-shell macadamia samples were divided into 17 batches of 200 nut-in-shell samples and placed into mesh bags. Nut-in-shell were either 1) kept wet at high temperatures for 5 days to induce brown centre formation and then dried, or 2) dried immediately using the Australian Macadamia Society (AMS) regime of 2 days at 37 °C, 2 days at 45 °C and 2 days at 54 °C [13]. The AMS regime is recommended as the industry best practice for drying macadamia nuts and is widely used to reduce the moisture concentration to the acceptable value of 1.5 % [23]. Thus, we created four treatments including: 1) brown centres from wet samples, (induced brown centres); 2) white centres from wet samples, (induced white centres); 3) brown centres from AMS dried samples (brown centres AMS); and 4) white centres from AMS dried samples (white centres AMS). Out of 17 bags, 8 bags were selected for the AMS drying regime. The remaining 9 bags were enclosed into oven proof plastic bags, 100 mL of water was added, and bags were sealed. The sealed bags were then placed into an oven at 40 °C for five days to induce brown centres. The nut-in-shell moisture concentration is at equilibrium at around 20 % over the storage period when ambient humidity is over 90 % [24]. The initial nut-in-shell moisture concentration was approximately 20 % and we added 100 mL of water to the bags and sealed the bags. This treatment produced a high ambient humidity of over 90 % throughout storage, with nut-in-shell moisture concentration maintained at approximately 20 %. After five days, the plastic bags were removed and bags of nut-in-shell samples were dried immediately using AMS regime of 2 days at 37 °C, 2 days at 45 °C and 2 days at 54 °C [13].

All nut-in-shell samples in each bag were cracked manually and each kernel was visually inspected to classify kernels with brown centres and white macadamia kernels. Kernels with a visible brown region in the centre of at least 7 mm in diameter were classified as brown centres. White macadamia kernels had no visible colour defects. The number of macadamia kernels with brown centres in each replicate was recorded after visual inspection.

**Fig. 1:**
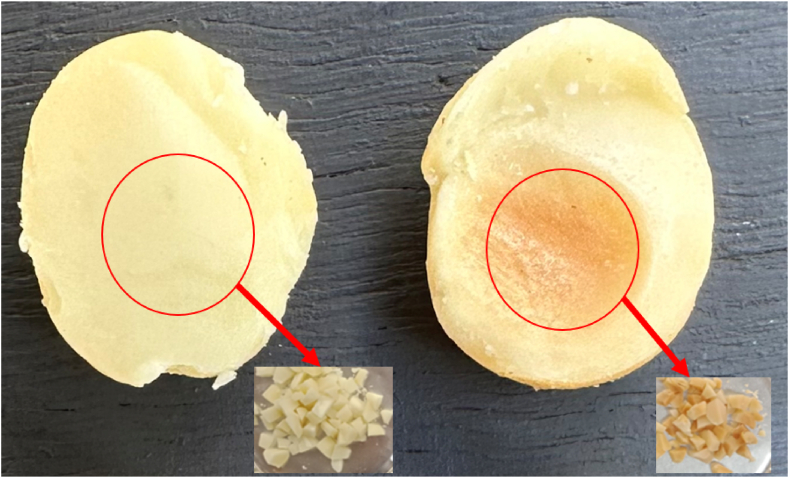
White centres and brown centres of macadamia kernels being cut out and used for chemical analysis.

We then randomly selected on average five kernels with brown centres and five white macadamia kernels from each replicate. After selecting brown centre and white centre kernels, the brown sections were cut and separated from the light and cream sections. The cut sections were divided into subsamples to assess kernel moisture concentration and chemical compositions. White macadamia kernels were also cut from the same region of the kernel for chemical analysis. Dissected samples from each batch were pooled to constitute one brown centre and one white centre sample for each bag (Fig. 1).

### 2.2 Moisture and chemical analysis of kernels

We measured moisture concentration of both nut-in-shell and kernel centres immediately after harvest, after storing wet for 5 days (induced) and after drying using the AMS regime [24]. We sampled five nut-in-shell samples from each bag to determine nut-in-shell moisture concentration. We measured kernel moisture concentration only once after drying nut-in-shell at the end of the experiment. We randomly selected a sub-sample of approximately 2 g of cut kernels from the cut section of each replicate to determine moisture concentration. We measured moisture concentrations using the thermogravimetric method [25]. We weighed nut-in-shell or kernel centre samples and placed them into an oven at 105 °C and reweighed them after 24 h. The moisture concentration of nut-in-shell and kernel centre samples was expressed as a percentage on a wet basis and was obtained using Equation (1):

(Equation 1) $$\text{MC}\;\left(\%\right)=\frac{\left(w-d\right)}{w}\times 100$$

where MC = moisture concentration expressed as a percentage, w = weight of sample before drying (g), d = weight of sample after drying (g).

The extraction of sugar and fat from macadamia kernel centres were carried out by mixing 1 g of macadamia kernel with 4 mL of methanol and then adding to each sample 2 mL of dichloromethane and 0.4 mL of deionised water. After mixing, 4 mL of dichloromethane and 2 mL of deionised water were added and then the mixture was centrifuged for 20 min to facilitate the separation of layers. The top layer was separated and transferred to a 10 mL tube to be dried under nitrogen flow 60 °C to remove the organic solvents and then in an oven at 60 °C to remove the water. The dried top layer was mixed with 1 mL of water and 1 mL of chloroform and then centrifuged to facilitate the separation of the layers again. The top aqueous layer was filtered and diluted (1:10) for sugar determination [26].

The remaining bottom organic layers were pooled and filtered into an 8 mL vial to be dried under nitrogen flow and then in an oven at 60 °C to extract the oil in the samples. After drying, the oil extracted was used to determine fatty acid methyl esters (FAME) and peroxide values.

### 2.3 Determination of kernel chemistry

#### 2.3.1 Sugar determination

The top aqueous layer previously extracted and prepared for sugar determination was analysed by means of high-pressure liquid chromatography (HPLC). Samples were injected into a Shimadzu NexeraX2 LC-30AD HPLC system (Shimadzu, Japan) with premixed HPLC grade water and acetonitrile as the mobile phase. A Luna ® Omega 3 μm SUGAR 100 Å, 100 × 4.6 mm column was used as the stationary phase (Phenomenex LTD; Aschaffenburg, Germany) following by a Shimadzu ELSD-LT II detector. Sugar peaks were identified by comparison with a known standard curve. Sugar concentrations were expressed as weight concentration (%w/w) of glucose, fructose and sucrose [22].

#### 2.3.2 Fatty acid methyl esters (FAME) determination

The composition of the fatty acid methyl esters (FAME) were determined by gas chromatography-mass spectrometry (GC-MS) analysis [27]. The previously extracted oil was diluted with chloroform to a final concentration of 10 mg/mL. Then, 50 μL of the oil solution was mixed with 50 mL of heneicosanoic acid (2 mg/mL in 1:1 chloroform/methanol) and 500 μL of 5 % acetyl chloride in methanol. The mixture was heated at 95 °C for 1 h and then allowed to cool down to room temperature. After cooling down, 500 μL of isooctane and 50 μL of 0.9 % sodium chloride solution were added and stirred vigorously. The mixture then was allowed to stand to allow layers to form again. Finally, 45 μL of the top isooctane layer was collected and transferred to a 250 μL glass insert with 45 μL of isooctane and 10 μL of methyl nonadecanoate (1 mg/mL in isooctane).

The FAME solution was then injected into a Shimadzu GCMS-QP2010 ULTRA equipped with a TRACE TR-FAME column (60 m × 0.25 mm (ID) × 0.25 μm; Thermo Fisher Scientific, Waltham, MA, USA). Individual FAME were identified by comparison of the sample peak retention times and mass spectrum against the NIST14 library and SUPELCO 37-component FAME mix standards. FAME were expressed as percentage of total fatty acids [27,28]. A chromatogram for a random kernel sample has been provided in Supplementary Fig. 1.

#### 2.3.3 Peroxide values detection

Peroxide values were determined by AOAC official method Cd 8-53 [29] with an adaptation for small size samples. Oil samples were dissolved in an acetic acid and chloroform solution (3:2). Then, 0.05 mL of potassium iodide was added and the solution was shaken for 1 min before distilled water and 1 % starch solution was added. The iodide ions in the solution were oxidised to iodine by the active oxygen (peroxides) in the product. The liberated iodine in the resulting mixture was titrated with standardised sodium thiosulfate. The volume of sodium thiosulfate was recorded for each sample. A sample blank was analysed similarly. The peroxide value was obtained using equation (2) and presented as meq. O_2_ kg^−1^ oil:

(Equation 2) $$\text{PV}=\frac{\left(S-B\right)\times N\times 1000}{W\times 1000}$$

where S = volume (μL) sodium thiosulfate used to titrate the sample, B = volume (μL) sodium thiosulfate used to titrate the blank, N = normality of sodium thiosulphate and W = weight of sample (g).

#### 2.3.4 Chemical composition analysis

Carbon, nitrogen and sulphur concentrations were determined using a combustion method. Macadamia kernel samples (0.25 g) were weighed in a ceramic boat and then placed into an induction furnace of a LECO 928 analyser. Samples were combusted at 1200 °C to convert the carbon and sulphur into carbon dioxide (CO_2_) and sulphur dioxide (SO_2_) which were then measured with infrared detention cells [30]. The nitrogen presented in the samples was combusted to nitrogen (N_2_), nitrogen dioxide (NO_2_) and nitrogen oxide (NO) gases and then reduced to N_2_ gas. N_2_ was determined using a thermal conductivity detector [31,32]. Carbon and nitrogen concentrations were expressed as weight percentage.

The remaining elements including aluminium, boron, calcium, cupper, iron, potassium, magnesium, manganese, sodium and zinc concentrations were determined using an acid digestion method. Macadamia kernel samples (0.25 g) were weighed and transferred into tubes to be mixed with an acid solution of 15 mL nitric/perchloric (5:1). The mixture was heated at 120 °C for approximately 1 h and then at 150 °C for another 1 h until the digestion was completed. The digested sample was made up to a volume of 25 mL and then analysed using a Perkin-Elmer Optima 8300 ICPOES for elemental concentration determination [28,32].

#### 2.3.5 Volatile compound determination

Volatile compounds of macadamia kernel samples were analysed via solid-phase microextraction (SPME) injection. Macadamia kernel samples (0.5 g) were placed into 20 mL solid phase micro extraction (SPME) autosampler vials and then incubated for 15 min at 60 °C with mixing at 500 rpm. After incubation, samples were exposed to a 2 cm 50/30 μm DVB/CAR/PDMS SPME fibre (Supelco, Inc., Bellefonte, PA) over the headspace for 30 min. The SPME needle was immediately injected into a Shimadzu GC-QP2010 ULTRA™ gas chromatography system (Shimadzu Corporation, Kyoto, Japan) with MS detector. A Thermo Scientific TR-FAME column (60 m Length, 0.25 mm ID, 0.25 μm film) was used to separate compounds for detection. Volatile compounds were identified by comparing the peaks of the mass spectrum against the NIST14 library. A chromatogram for a random kernel sample has been provided in Supplementary Fig. 1.

### 2.4 Statistical analysis

We tested for differences in chemical measures between induced brown centres, induced white centres, AMS brown centres and AMS white centres using a series of one-way ANOVAs. We tested for differences in moisture concentration, sugar concentration, fatty acid composition, peroxide value and nutrient concentrations amongst treatments. All data displayed a Gaussian distribution. The ANOVAs were followed by Tukey HSD tests when significant differences were observed (p < 0.05). All data presented in tables and figures are mean ± standard errors. All statistical analyses were performed using IMB SPSS Statistics v. 27.

## 3 Results

### 3.1 Brown centre occurrence and moisture concentration of nut-in-shell and kernel samples

**Fig. 2:**
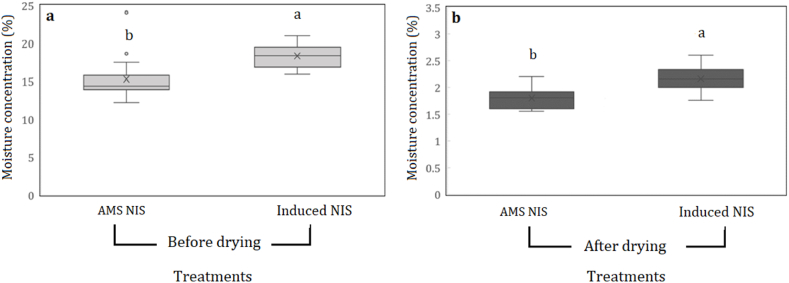
Differences (mean ± standard errors) in moisture concentration (%) of nut-in-shell (NIS) between induced NIS and AMS NIS. Moisture concentration of NIS was determined before (a) and after (b) drying for both induced and AMS samples. Induced NIS were stored at high moisture concentration at 40 °C for five days and then dried using the AMS drying regime. AMS samples were dried immediately after harvest using the AMS drying regime (AMS NIS). Treatments with different letters are significantly different at p < 0.05.

**Fig. 3:**
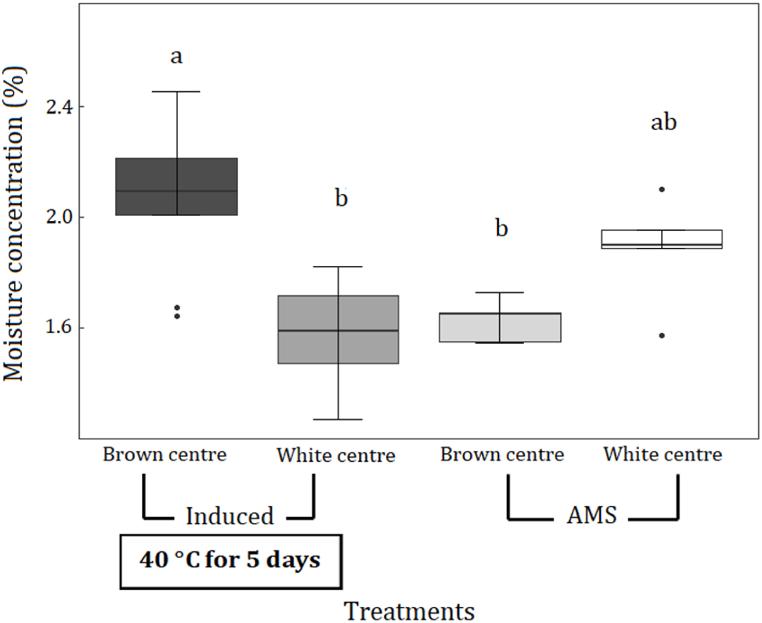
Differences (mean ± standard errors) in moisture concentration (%) of brown centres and white centres between induced nut-in-shell (NIS) and AMS dried nut-in-shell (NIS) samples. Induced NIS were stored at high moisture concentration at 40 °C for five days and then dried using the AMS drying regime (Induced). AMS samples were dried immediately after harvest using the AMS drying regime (AMS). Treatments with different letters are significantly different at p < 0.05.

We found macadamia kernels developed significantly more brown centres when induced than those dried under the AMS regime (10.33 % ± 0.62 vs 1.44 % ± 0.33). We also found that the moisture concentration of nut-in-shell was higher in induced samples compared with that of AMS samples immediately dried after harvest (Fig. 2a). Similarly, moisture concentration after drying was higher in induced nut-in-shell (2.16 %) compared with AMS samples being immediately dried (1.80 %) (Fig. 2b). Induced brown centres had a significantly higher moisture concentration (2.07 %) compared with induced white centres (1.56 %) (Fig. 3). In contrast, we did not find any significant differences between moisture concentration of AMS brown centres and AMS white centres.

### 3.2 Chemical composition

**Fig. 4:**
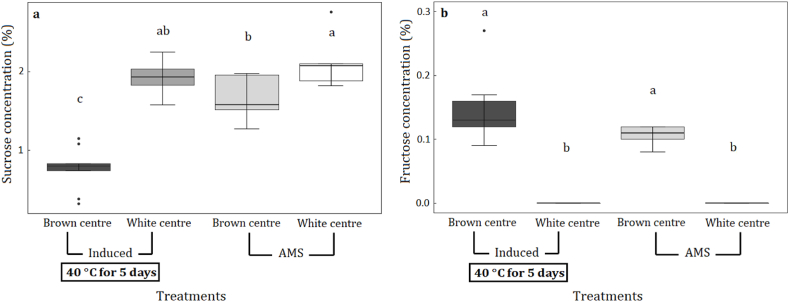
Differences (mean ± standard errors) in sucrose (**a**) and fructose (**b**) concentration (%) of brown centres and white centres among induced nut-in-shell (NIS) and AMS dried nut-in-shell (NIS) samples. Induced NIS were stored at high moisture concentration at 40 °C for five days and then dried using the AMS drying regime (Induced). AMS samples were dried immediately after harvest using the AMS drying regime (AMS). Treatments with different letters are significantly different at p < 0.05.

Although glucose was not detected in any sample, we did find that induced brown centres contained approximately 60 % lower sucrose concentration than induced white centres (Fig. 4a). Similarly, sucrose concentrations in AMS brown centre samples were significantly lower than in AMS white centres (Fig. 4a). In addition, fructose was present in brown centres of both induced and AMS samples but was not detected in white centres (Fig. 4b).

We did not find any significant differences in the peroxide values and fatty acid compositions across all treatments (Table 1). Peroxide values were 0.18 ± 0.03 and 0.22 ± 0.04 meq O_2_/kg oil in brown and white centres of induced samples, respectively, and 0.28 ± 0.03 and 0.20 ± 0.04 meq O_2_/kg oil in brown and white centres of AMS samples, respectively. Total unsaturated fatty acid concentration of all treatments was in the range of 82.49 % and 83.80 % and saturated fatty acid concentration in the range of 16.19 % and 17.51 % (Table 1).

**Table 1:** Differences (mean ± standard errors) in fatty acid concentration (%) of brown centres and white centres among induced nut-in-shell (NIS) and AMS dried nut-in-shell (NIS) samples. Induced brown centres and white centres were stored at high moisture content at 40 °C for five days and then dried using AMS drying regime. AMS brown centres and white centres were dried immediately after harvest using AMS drying regime. Treatments with no letters are not significantly different at p < 0.05

Fatty acid	Relative abundance of fatty acid (%)							
	Induced brown centre		Induced white centre		AMS brown centre		AMS white centre	
C14:0 (Myristic acid)	0.74	±0.40	0.73	±0.20	0.77	±0.08	0.61	±0.21
C16:0 (Palmitic acid)	9.61	±0.23	9.91	±0.20	9.20	±0.32	9.90	±0.30
C16:1n-7 (Palmitoleic acid)	15.51	±0.63	15.03	±0.40	13.61	±0.37	13.58	±0.52
C18:0 (Stearic acid)	2.98	±0.14	2.94	±0.10	2.86	±0.19	3.38	±0.81
C18:1n-9 (Oleic acid)	64.28	±0.66	64.22	±0.37	66.12	±0.60	65.12	±0.74
C18:2n-6 (Linoleic acid)	1.39	±0.08	1.62	±0.06	1.82	±0.33	1.48	±0.12
C20:0 (Arachidic acid)	2.55	±0.06	2.55	±0.10	2.59	±0.13	2.82	±0.04
C20:1n-9 (Gondoic acid)	2.05	±0.50	2.11	±0.06	2.12	±0.10	2.15	±0.04
C22:0 (Behenic acid)	0.52	±0.02	0.51	±0.03	0.51	±0.04	0.55	±0.01
C22:1n-9 (Erucic acid)	0.12	±0.01	0.13	±0.01	0.14	±0.01	0.15	±0.01
C24:0 (Lignoceric acid)	0.23	±0.10	0.25	±0.02	0.27	±0.04	0.25	±0.01
Total unsaturated fatty acids	83.36	±0.33	83.11	±0.41	83.80	±0.56	82.49	±0.41
Total saturated fatty acids	16.64	±0.33	16.89	±0.41	16.19	±0.56	17.51	±0.41

**Fig. 5:**
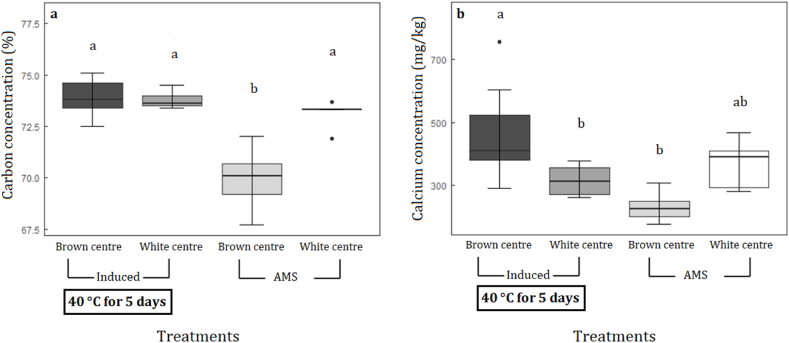
Differences (mean ± standard errors) in carbon (a) and calcium (b) concentrations (%) of brown centres and white centres among induced nut-in-shell (NIS) and AMS dried nut-in-shell (NIS) samples. Induced NIS were stored at high moisture concentration at 40 °C for five days and then dried using the AMS drying regime (Induced). AMS samples were dried immediately after harvest using the AMS drying regime (AMS). Treatments with different letters are significantly different at p < 0.05.

Most of the nutrient concentrations were not significantly different amongst treatments (Table 2). We found that carbon concentrations were significantly lower in AMS brown centres compared with all other treatments (Fig. 5a). Furthermore, calcium concentrations were significantly higher in induced brown centres compared with induced white centres. Induced brown centres had a 33 % higher calcium concentration than induced white centre kernel (Fig. 5b). Phosphorus concentrations of the induced samples was 15 % higher in brown centres than white centres. However, calcium and phosphorus concentrations were not significantly different between AMS brown centres and AMS white centres (Table 2).

**Table 2:** Differences (mean ± standard errors) in nutrient concentrations between brown centres and white centres among induced nut-in-shell (NIS) and AMS dried nut-in-shell (NIS) samples. Induced brown centres and white centres were stored at high moisture content at 40 °C for five days and then dried using AMS drying regime. AMS brown centres and white centres were dried immediately after harvest using AMS drying regime. Treatments with no letters are not significantly different at p < 0.05

Nitrogen (%)	Induced Brown Centre		Induced White Centre		AMS Brown Centre		AMS White Centre	
	1.45	±0.03	1.40	±0.19	1.50	±0.37	1.49	±0.19
Aluminium (mg/kg)	18.42	±2.23	15.06	±1.09	17.96	±3.89	17.54	±0.86
Boron (mg/kg)	22.29	±2.11	19.08	±1.45	22.68	±3.80	23.72	±3.44
Copper (mg/kg)	6.26	±2.39	4.58	±0.15	3.96	±0.62	5.96	±2.44
Iron (mg/kg)	11.23	±0.33	11.37	±0.33	10.54	±0.80	12.62	±1.05
Potassium (%)	0.49	±0.02	0.48	±0.01	0.56	±0.04	0.47	±0.44
Magnesium (%)	0.17	±0.01	0.15	±0.00	0.18	±0.00	0.16	±0.01
Manganese (mg/kg)	5.47	±1.05 ab	5.67	±0.70a	2.62	±0.72b	3.56	±0.40 ab
Sodium (mg/kg)	53.17	±9.45	68.63	±9.98	100.56	±42.03	53.84	±7.81
Phosphorus (%)	0.26	±0.01a	0.22	±0.01b	0.26	±0.03 ab	0.23	±0.01 ab
Sulphur (%)	0.15	±0.01 ab	0.13	±0.00b	0.15	±0.01a	0.14	±0.00 ab
Zinc (mg/kg)	15.92	±1.61	13.51	±0.70	14.40	±1.61	16.46	±1.66

### 3.3 Differences in volatile compounds between brown centre and white macadamia kernels

The main volatile compounds identified included hydrocarbons, esters, alcohols and acids (Table 3). The peak area of the hydrocarbon nonane was significantly lower in induced samples compared with AMS samples. Peak areas of nonanoic and octanoic acid were significantly lower in AMS white centres compared with all other treatments. The alcohol 2,3 butanediol was only detected in induced brown centres and induced white centres. Induced brown centres had a higher peak area of 2,3 butanediol compared with induced white centres. The peak area of 3-methoxy-3-methyl-1-butanol in both induced and AMS samples was lower in brown centres than white centres. The peak area of the ketone ethenone was not significantly different amongst all treatments.

**Table 3:** Differences (mean ± standard errors) in volatile compound concentrations (% peak area) of brown centres and white centres among induced nut-in-shell (NIS) and AMS dried nut-in-shell (NIS) samples. Induced brown centres and white centres were stored at high moisture content at 40 °C for five days and then dried using the AMS drying regime. AMS brown centres and white centres were dried immediately after harvest using the AMS drying regime. Treatments with different letters are significantly different at p < 0.05

Volatile compounds	Induced brown centre	Induced white centre	AMS brown centre	AMS white centre
2,4,7,9-Tetramethyl-5-decyn-4,7-diol	33.18 ± 0.99	41.95 ± 2.33	40.58 ± 3.33	42.04 ± 3.49
Undecane	4.06 ± 0.61 ab	3.27 ± 0.50b	7.59 ± 1.90a	5.87 ± 1.15 ab
Dodecane	3.14 ± 0.47	2.03 ± 0.46	3.60 ± 1.86	5.53 ± 1.30
Nonane	2.23 ± 0.27b	2.48 ± 0.61b	5.14 ± 0.97a	7.27 ± 0.51a
Octane	0.25 ± 0.06	0.49 ± 0.21	0.63 ± 0.30	0.30 ± 0.24
Total hydrocarbons	11.26 ± 1.21bc	9.39 ± 1.23c	19.10 ± 3.92 ab	20.77 ± 1.92a
Nonanoic acid	21.49 ± 1.01b	16.78 ± 2.49b	16.21 ± 1.77b	3.60 ± 0.48a
Octanoic acid	4.38 ± 0.41a	3.14 ± 0.43a	3.47 ± 0.60a	1.03 ± 0.33b
Total Acids	25.90 ± 1.35a	19.93 ± 2.70a	19.68 ± 2.05a	4.63 ± 0.69b
2,3 Butaneidol	13.56 ± 1.03a	6.12 ± 1.59b	0 ± 0.00c	0 ± 0.00c
3 methoxy 3 methyl butanol	5.67 ± 0.48b	10.63 ± 0.95a	6.88 ± 0.83b	11.88 ± 1.54a
Total Alcohols	19.22 ± 1.14a	16.75 ± 1.67 ab	6.88 ± 0.83c	11.88 ± 1.54b
1,2 - Benzenedicaboxylic acid ester	0.91 ± 0.16b	3.19 ± 0.61a	2.51 ± 0.42 ab	3.27 ± 0.45a
Total Esters	1.67 ± 0.22b	4.22 ± 0.80a	4.75 ± 0.56a	5.59 ± 1.07a
Butylated Hydroxytoluene	1.73 ± 0.18b	1.33 ± 0.15b	2.13 ± 0.60b	3.34 ± 0.23a
2,2,4-Trimethyl-1,3-pentanediol diisobutyrate	0.79 ± 0.07b	0.88 ± 0.80b	1.29 ± 0.25	1.67 ± 0.70a
Ethanone, 1-(4,5-diethyl-2-methyl-1-cyclopenten-1-yl)-	0.28 ± 0.05	0.18 ± 0.09	0.28 ± 0.08	0.07 ± 0.05
2H-1-Benzopyran, 3,5,6,8a-tetrahydro-2,5,5,8a-tetramethyl-, trans-	0.91 ± 0.26 ab	1.70 ± 0.36 ab	0.09 ± 0.06b	2.51 0.88a

## 4 Discussion

We found that nut-in-shell samples stored under wet conditions at 40 °C and then dried using the AMS regime had higher moisture concentration in their kernels than those nut-in-shell samples dried immediately using the AMS regime after harvest. Storing macadamia nut-in-shell under wet conditions at 40 °C also greatly increased brown centre formation to 10.33 %. Our findings are consistent with another study where storing nut-in-shell macadamia samples under the same conditions as of our study has resulted in the formation of brown centres in 9.6 % of kernels [13]. These findings imply that the formation of brown centres strongly depends on moisture concentration and temperature, and that proper storage conditions are fundamental to avoid the formation of brown centre in raw macadamia kernels [13]. We also found that induced brown centres had a higher moisture concentration even after drying using the AMS protocol, compared with white centres of macadamia kernels kept under wet conditions prior to drying. Macadamia kernels with moisture concentrations over the acceptable limits of 1.5 % are prone to quality deterioration, reduction of the shelf life and spoilage [5,9,33]. The induced brown centres had a moisture concentration of 2.07 % and hence are susceptible to decreased quality and shelf life.

Induced brown centres had lower sucrose and higher fructose concentrations than induced white centres. Similarly, brown centres of AMS samples had lower sucrose and higher fructose concentrations compared with the counterpart AMS white centres. In both postharvest conditions (induced and AMS conditions), the same pattern in sugar concentration was observed, suggesting sugar hydrolysis could be one of the main factors leading the formation of brown centres in macadamia kernels, both in laboratory induced samples and those occurring under standard industry postharvest drying regimes. Roasted hazelnuts with internal browning have higher total sugar and sucrose concentrations compared with white hazelnuts [34]. The breakdown of sucrose to reducing sugars (e.g. fructose and glucose) is initiated by elevated moisture and heat [5,12,13]. Reducing sugars are degraded, resulting in the initiation of the Maillard reaction to form brown colouration in nut kernels [10,12,34]. The incidence of brown centres in induced samples could be partly explained by hydrolysis of sucrose to reducing sugars under high moisture concentration [12]. In AMS samples, the formation of brown centres was also associated with sucrose hydrolysis, however, the moisture concentration of brown centres in AMS samples were not as high as those formed under wet and hot conditions. In our study, brown centre formation could be mainly explained by hydrolysis of sucrose to reducing sugars initiating the Maillard reaction.

Calcium and phosphorus concentrations were significantly higher in induced brown centres compared with induced white centres but did not differ between AMS brown centres and AMS white centres. Excessive calcium concentration in cell walls has led to skin discoloration and salt damage in fruits, but it is uncertain how excessive calcium concentration in cell walls can affect browning in nuts [35]. In our study, we determined total calcium concentration rather than calcium concentration in cell walls. We also found high within-treatment variation among samples which may have been caused by pre-harvest management practices. These results were unexpected because calcium deficiencies have been mainly associated with internal discoloration in other nuts [8,11,36]. However, we observed a higher calcium concentration in the induced brown centres compared with induced white centres. Nutrient concentrations in macadamia kernel could be controlled by preharvest fertilisation [37]. However, our results of nutrient concentration differences between brown and white sections of the macadamia were not very conclusive. Hence, nutrient differences as a factor to initiate brown centre still remained unclear, but forming brown centres in macadamia kernels are not likely to be driven calcium deficiency.

We also found no significant differences in fatty acid compositions/concentrations and peroxide values between brown centres and white centres of both induced and AMS samples. Oil oxidation has been associated with internal discolouration in other nuts [10]. Some volatile compounds (e.g., nonanoic and octanoic acid) have been also identified as potential markers of lipid oxidation in roasted almonds after 20 weeks of storage at 35 °C and 65 % relative humidity [38]. We found that both nonanoic and octanoic acid were significantly higher in induced and AMS brown centres compared with AMS white centres, indicating that the oil degradation was initiated, although it was not yet evident in the peroxide values. The lack of differences in peroxide values suggests that peroxide value was not sensitive enough to study lipid oxidation in brown centres.

We also only detected 2,3 butanediol in induced samples, and induced brown centres had higher peak area of 2,3 butanediol than induced white centres. The presence of 2,3 butanediol in almond has been related to almond kernels being exposed to high moisture concentrations of above 5 % [10]. In macadamia kernels, the detection of 2,3 butanediol and 3-hydroxy-2-butanone has been associated with grey kernels infected with *Enterobacter cloacae* [39]. *E. cloacae* is a species of bacteria capable of spoilage in nuts and can cause off-odours and/or off-flavours in food [39]. Bacterial infection was not surprising to be detected when brown centre samples had high moisture concentration which provides an optimal growing condition for bacteria.

Our study suggests that improper postharvest handling when high humidity and elevated temperatures occur in orchards or silos contribute to brown centre formation in macadamia kernels. For example, storing wet nuts in silos with poor ventilation has increased brown centre formation previously [9]. Thus, monitoring weather to ensure nuts are harvested before wet and humid conditions, fast tracking nut processing after harvest, and improving air flow in silos would minimise brown centre formation. The variability introduced by variety and environment has been identified as a shortfall in our previous study where the results may have been masked with high within sample variability [15]. We used a single mid-late season variety of macadamia collected from one orchard to remove the variability introduced by variety and environment. This study, hence, needs to be extended to examine other varieties from various orchards. Macadamia cultivars also produce nuts with variable sizes and shell thickness. Thus, future studies need to explore to what extent early and late season cultivars differ in their susceptibility to form brown centre kernels under high temperature and to understand how nut-in-shell characteristics such as size and shell thickness influence brown centre formation.

## 5 Conclusion

Our research confirmed that storage of macadamia nut-in-shell at high temperatures and humidity increased brown centre incidences in macadamia kernels. Furthermore, our results imply that sugar hydrolysis and the Maillard reaction is associated with brown centres both in laboratory induced samples and those formed using industry best practice drying methods. The volatile compounds also suggested an initiation of oil oxidation and potential bacterial infection in brown centre formation. Our study suggests that improper postharvest handling is contributing to brown centre formation in macadamia kernels. In particular, improper drying and storage at high temperature and humidity are likely to result in sugar hydrolysis and brown centre formation. We recommend storing macadamia nut-in-shell under appropriate temperatures (below 30 °C) and low relative humidity to reduce brown centre formation.

## Data availability statement

Data will be made available on request.

## Additional information

No additional information is available for this paper.

## CRediT authorship contribution statement

**Marcela Martinez:** Writing – review & editing, Writing – original draft, Methodology, Investigation, Formal analysis, Data curation, Conceptualization. **Helen M. Wallace:** Writing – review & editing, Validation, Supervision, Funding acquisition, Conceptualization. **Chris Searle:** Writing – review & editing, Resources, Conceptualization. **Brittany Elliott:** Writing – review & editing, Visualization, Validation. **Shahla Hosseini Bai:** Writing – review & editing, Validation, Supervision, Project administration, Investigation, Formal analysis, Conceptualization.

## Declaration of competing interest

The authors declare the following financial interests/personal relationships which may be considered as potential competing interests:Shahla Hosseini Bai - Helen Wallace reports financial support was provided by Macadamia processors including Hinkler Park Plantations, Macadamias Australia, RFM Macadamias and TQ Holdings Pty Ltd. The authors would like to declare that co-author Chris Searle works as a consultant for the Macadamia industry, through the business MacAvo Consulting, however this consulting relationship had no influence on the research undertaken in this manuscript. The financial supporters had no influence to interpret data involved in this research, or in the writing of this manuscript.
