# Supplementary material for: Understanding chemical pathways of brown centre formation in laboratory induced and conventionally dried nut-in-shell macadamia kernels
Source: Heliyon. 2024 Jan 30;10(3):e25221. doi: 10.1016/j.heliyon.2024.e25221 (PMC10864918; doi:10.1016/j.heliyon.2024.e25221)
Supplement: Multimedia component 1 [file mmc1.docx]

**Supplementary Figure**

| 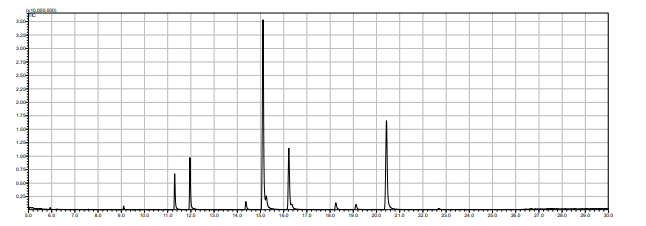  a |
| --- |
| 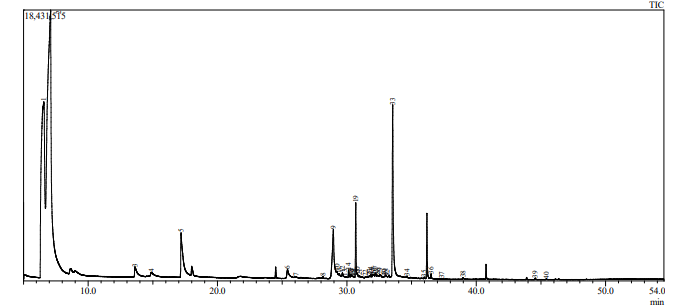  b |

**Figure S1:** GC-MS chromatograms of a random kernel sample for fatty acid methyl esters (a) and volatile compounds (b).
